# 12/15-lipoxygenase activity promotes efficient inflammation resolution in a murine model of Lyme arthritis

**DOI:** 10.3389/fimmu.2023.1144172

**Published:** 2023-04-18

**Authors:** Christa D. Jackson, Kinsey A. Hilliard, Charles R. Brown

**Affiliations:** Department of Veterinary Pathobiology, College of Veterinary Medicine, University of Missouri, Columbia, MO, United States

**Keywords:** Lyme disease, *Borrelia burgdorferi*, arthritis, resolution, eicosanoids, inflammation

## Abstract

Infection of C3H/HeJ (C3H) mice with *Borrelia burgdorferi* results in the development of a robust inflammatory arthritis that peaks around 3-4 weeks post-infection and then spontaneously resolves over the next few weeks. Mice lacking cyclooxygenase (COX)-2 or 5-lipoxygenase (5-LO) activity develop arthritis similar to wild-type mice but display delayed or prolonged joint resolution. Since 12/15-lipoxygenase (12/15-LO) activity is generally down-stream of both COX-2 and 5-LO activity and results in the production of pro-resolution lipids such as lipoxins and resolvins among others, we investigated the impact of 12/15-LO deficiency on the resolution of Lyme arthritis in mice on a C3H background. We found the expression of *Alox15* (12/15-LO gene) peaked around 4-weeks post-infection in C3H mice suggesting a role for 12/15-LO in mediating arthritis resolution. A deficiency in 12/15-LO resulted in exacerbated ankle swelling and arthritis severity during the resolution phase without compromising anti-*Borrelia* antibody production and spirochete clearance. However, clearance of inflammatory cells was impeded. Therapeutic treatment of *B. burgdorferi*-infected C3H mice with lipoxin A4 (LXA_4_) near the peak of disease resulted in significantly decreased ankle swelling and a switch of joint macrophages to a resolving phenotype but did not directly impact arthritis severity. These results demonstrate that 12/15-LO lipid metabolites are important components of inflammatory arthritis resolution in murine Lyme arthritis and may be a therapeutic target for treatment of joint edema and pain for Lyme arthritis patients without compromising spirochete clearance.

## Introduction

Lyme disease is caused by infection with the spirochete *Borrelia burgdorferi* transmitted via the bite of an infected *Ixodes* tick (1). A Centers for Disease Control and Prevention report estimated that 476,000 Americans are treated for Lyme disease each year (2), making Lyme disease the most common vector-borne disease in the United States. Early antibiotic treatment is largely effective at controlling infection and preventing further disease progression. However, without early treatment, *B. burgdorferi* can disseminate and cause debilitating sequelae including cranial neuropathy, carditis, and arthritis. Approximately 62-80% of patients with disseminated infection develop joint pain and arthritis (3), and a small percentage of these patients may have a recurring or persistent inflammatory arthritis even after antibiotic treatment. As these patients present with a recurring Lyme arthritis despite no evident ongoing infection, this condition is referred to as postinfectious Lyme arthritis (pLA) (4).

It is now understood that inflammation does not passively resolve after pathogen clearance, but requires pro-resolution mechanisms to be actively initiated for successful control of inflammation (5). Both protein and bioactive lipid molecules are critical components of inflammation regulation, and much work has been done to identify and characterize their roles in models of inflammatory disease. Examples of bioactive lipid mediators include eicosanoids and specialized pro-resolving mediators (SPM). Eicosanoids are arachidonic acid (AA)-derived lipids that play a role in both the induction and resolution of inflammation (6), whereas SPM are a functional class of lipids derived from omega-3 fatty acids such as docosahexaeneoic acid (DHA) or eicosapentaenoic acid (EPA) that are characterized by pro-resolving activity in a number of disease models (7). The temporal regulation of bioactive lipid class-switching from proinflammatory to proresolving activity is critical to the timely promotion of tissue healing and restoration of homeostasis (8).

In the murine model of Lyme arthritis (mLA), arthritis-susceptible C3H/HeJ mice (9) infected with *B. burgdorferi* develop an inflammatory arthritis characterized by robust inflammatory cell infiltrate and synovial hyperplasia in the tibiotarsal joint, followed by spontaneous resolution starting around three to four weeks post-infection (pi) (10). While mLA resolution correlates with antibody-mediated spirochete clearance from the joint (9), disruption of eicosanoid production or signaling has adverse effects on mLA resolution despite sufficient bacterial control, mimicking pLA in humans (11–13). Indeed, we previously demonstrated that pharmacological inhibition or genetic deletion of eicosanoid metabolic enzymes cyclooxygenase-2 (COX-2) or 5-lipoxygenase (5-LO) resulted in defective mLA resolution with persistent immune infiltrate (11, 12). A potential shared mechanism behind the resolution defects in these knockout mice strains lies in the ability of these enzymes and their metabolites to either cooperate with or regulate the enzyme 12/15-lipoxygenase (12/15-LO) to promote the synthesis of lipoxin A_4_ (LXA_4_) and other SPM (8, 14–16). Therefore, a breakdown in 12/15-LO induction or activity prevents production of these pro-resolution lipid metabolites and may contribute to the mLA resolution defects seen in COX-2^-/-^ and 5-LO^-/-^ mice.

To elucidate the role of 12/15-LO activity in mLA, we infected wild-type (WT) and 12/15-LO^-/-^ C3H mice with *B. burgdorferi* and characterized the development and resolution of arthritis in the tibiotarsal joints. While *B. burgdorferi*-infected 12/15-LO^-/-^ mice still developed a robust arthritis, they showed a defect in efficient resolution of arthritis despite successful bacterial control. This non-resolving phenotype was characterized by persistent neutrophil and macrophage populations in the joint even as this infiltrate was cleared in WT controls. *In vitro* experiments suggested that inflammatory cell persistence may be due to defective efferocytic removal of apoptotic 12/15-LO^-/-^ neutrophils by macrophages. To further investigate the contribution of 12/15-LO activity to mLA, we tested the ability of 12/15-LO metabolite LXA_4_ to induce arthritis resolution. Therapeutic treatment of mLA in WT C3H mice with exogenous LXA_4_ around the peak of inflammation reduced edema and remodeled joint macrophage populations towards a pro-resolving phenotype. Two weeks later, macrophage and neutrophil numbers were significantly decreased in LXA_4_-treated mice, though overall arthritis severity was not reduced. Together, these findings demonstrate that 12/15-LO production of lipoxins and SPM are critical for efficient mLA resolution and lipoxin treatment may be efficacious to reduce joint edema without compromising bacterial clearance.

## Materials and methods

### Animals

All mice are on a C3H/HeJ background. 12/15-LO knockout mice (B6.129S2-*Alox15^tm1Fun^*/J stock #002778) were purchased from The Jackson Laboratory (Bar Harbor, ME) and backcrossed to a C3H/HeJ background for at least ten generations in our colony. Approximately equal numbers of male and female mice were used for *in vivo* experiments and as the source of bone marrow-derived cells. Animals were housed in a specific pathogen-free facility and given sterile food and water ad libitum. All studies were conducted in accordance with the Animal Care and Use Committee of the University of Missouri.

### Bacteria and infections

A low-passage virulent N40 strain *B. burgdorferi* (17) was grown to log phase in complete Barbour-Stoenner-Kelly (BSK)-H media (Sigma, St. Louis, MO) at 32°C (18). For *in vivo* infections, 5x10^4^ spirochetes in 50μl incomplete BSK-H media (without rabbit serum) were injected into each hind footpad (19). For *in vitro* studies, *B. burgdorferi* was used at a multiplicity of infection (MOI) of 10 (19).

### Ankle swelling and arthritis assessment

Ankle swelling was monitored by measuring the thickest cranio-caudal portion of the tibiotarsal joint using a metric caliper. The baseline (0 dpi) values were subtracted from weekly measurements to determine the increase in ankle diameter (20). After euthanasia, one ankle from each mouse was harvested for histological staining. Histology samples were preserved in formalin and submitted to the University of Missouri Veterinary Medicine Diagnostic Lab (VMDL) for hematoxylin and eosin (H&E) staining. Sections were scored for arthritis severity on a scale of 0 to 4 as described (21). Briefly, a score of 0 represents no evident inflammatory cells, 1 represents 1-10% inflammatory cells, 2 represents 11-25%, 3 represents 26-50% and 4 represents inflammation involving more than 50% of the section. Sections were scored in a double-blind manner by two trained individuals and the average score of each sample was plotted.

### Cell isolation and flow cytometry

Harvested joints were digested in collagenase/dispase with DNase and shredded with rat-tooth forceps to isolate cells before staining them for flow cytometry as described (22). About 5x10^5^ cells were incubated in a 96-well U-bottom plate with Fc block (anti-CD16/CD32; eBioscience, San Diego, CA) then surface stained as indicated with the following antibodies: CD45.2 PE, F4/80 APC, Ly-6G PE-Cy7, and Ly-6C FITC (all from eBioscience). Cells were then washed and fixed in 4% paraformaldehyde for 15 minutes. For each sample 50,000 events were analyzed using a BD LSRFortessa X-20 flow cytometer and data analysis was performed using FlowJo 10.8.1 software.

### *B. burgdorferi* loads

DNA was isolated from harvested ankle tissue homogenized in TRIzol reagent according to manufacturer’s specifications (Invitrogen, Waltham, MA), followed by an ethanol precipitation step for DNA cleanup as needed. qPCR was performed using Power SYBR Green PCR Master Mix (Applied Biosystems, Waltham, MA) and results for *B. burgdorferi* flagellin (*flaB*) were normalized to mouse nidogen (*Nid1*). Bacterial loads are reported as copies of *flaB* per 1000 copies of *Nid1* (21).

### Determination of serum antibody levels

Blood was collected from experimental mice using cardiac puncture and serum was separated by gravity. *B. burgdorferi*-specific IgM and IgG levels in the sera of infected mice were then determined by enzyme-linked immunosorbent assay, as described (23).

### RNA isolation, cDNA generation, and qPCR

RNA was isolated from ankle tissue using TRIzol reagent according to manufacturer’s specifications (Invitrogen). RNA was isolated from *in vitro* cultures using a Qiagen RNeasy kit (Qiagen, Germantown, MD). cDNA was synthesized using a High-Capacity cDNA Reverse Transcription Kit (Applied Biosystems). qPCR was performed using Power SYBR Green PCR Master Mix (ThermoFischer Scientific, Waltham, MA) and target gene expression was calculated by -ΔΔCt compared to housekeeping genes *Nid1* for tissue samples or *Gapdh* for *in vitro* experiments. Relative expression was calculated as log_2_ fold change from D0 (uninfected) tissue samples or unstimulated BMDM. Primers used are listed in **Supplemental Table 1**.

### Bone marrow-derived macrophage (BMDM) generation

Bone marrow was isolated from mouse tibias and femurs and allowed to differentiate for 6 days in DMEM supplemented with 30% L929 cell-conditioned medium, 10% FBS and 1% penicillin/streptomycin (P/S) at 37°C in 5% CO_2_, with media replacement on day 3. Adherent cells were scraped, washed, and plated to adhere overnight at 37°C in 5% CO_2_ before use.

### Bone marrow neutrophil (BMN) isolation and apoptosis

Bone marrow was isolated from mouse tibias and femurs and enriched for neutrophils by separation on a 2-step Histopaque gradient (1.119 g/ml and 1.083 g/ml; MilliporeSigma, Burlington, MA) at 700x*g* for 30 minutes at RT as described (24). ACK lysis buffer treatment was used to remove contaminating red blood cells. Cells were then resuspended in RPMI 1640 with 10% FBS and 1% P/S, plated, and incubated for 24 hr at 37°C in 5% CO_2_ to allow spontaneous apoptosis. Apoptosis was determined using the PE Annexin V Apoptosis Detection Kit I (BD Biosciences, San Jose, CA).

### Efferocytosis assay

BMN were harvested as described above, labeled with CellTrace Far Red Cell Proliferation Kit (APC-Cy7) (ThermoFisher Scientific), and rested for 24 hours at 37°C in 5% CO_2_ to allow spontaneous apoptosis. Labeled apoptotic BMN (AN) were added to BMDM (2:1) in a 24-well plate and incubated for 4 hours at 37°C in 5% CO_2_. Cells were then washed and stained with CD45.2 PE and F4/80 APC (both from eBioscience) for flow cytometry with 50,000 events collected per sample. Efferocytosis was determined to have occurred in cells which were CD45.2^+^F4/80^+^APC-Cy7^+^.

### Lipid quantification

WT or 12/15-LO^-/-^ BMDM were cultured with or without *B. burgdorferi* (MOI 10) 2 hours, after which supernatant was harvested and run on a LXA_4_ EIA kit (Cayman Chemical, Ann Arbor, MI).

### LXA_4_ treatment

For *in vivo* experiments, WT C3H mice were treated intraperitoneally (i.p.) with 100ul of either vehicle control (10% EtOH in PBS) or 1μg LXA_4_ (Cayman Chemical) (25, 26) in PBS on 18, 19, and 20 dpi.

### Statistics

Data shown is representative of at least two independent experiments. Where indicated, data from two experiments are combined to reach power. Data is shown as mean +/- standard deviation unless otherwise noted. Data consisting of two groups were compared using an unpaired, two-tailed Student’s t test. Significance among three or more groups was assessed by one-way ANOVA with Tukey’s post-test unless the groups were all compared to a single control, in which case Dunnett’s test was used instead. Statistical significance of histology scores was determined by a nonparametric Mann-Whitney U test when between two groups and by Kruskal-Wallis one-way ANOVA when between three or more groups. Significance was set at p<0.05 for all tests.

## Results

### mLA resolution is defective in C3H 12/15-LO^-/-^ mice

**Figure 1:**
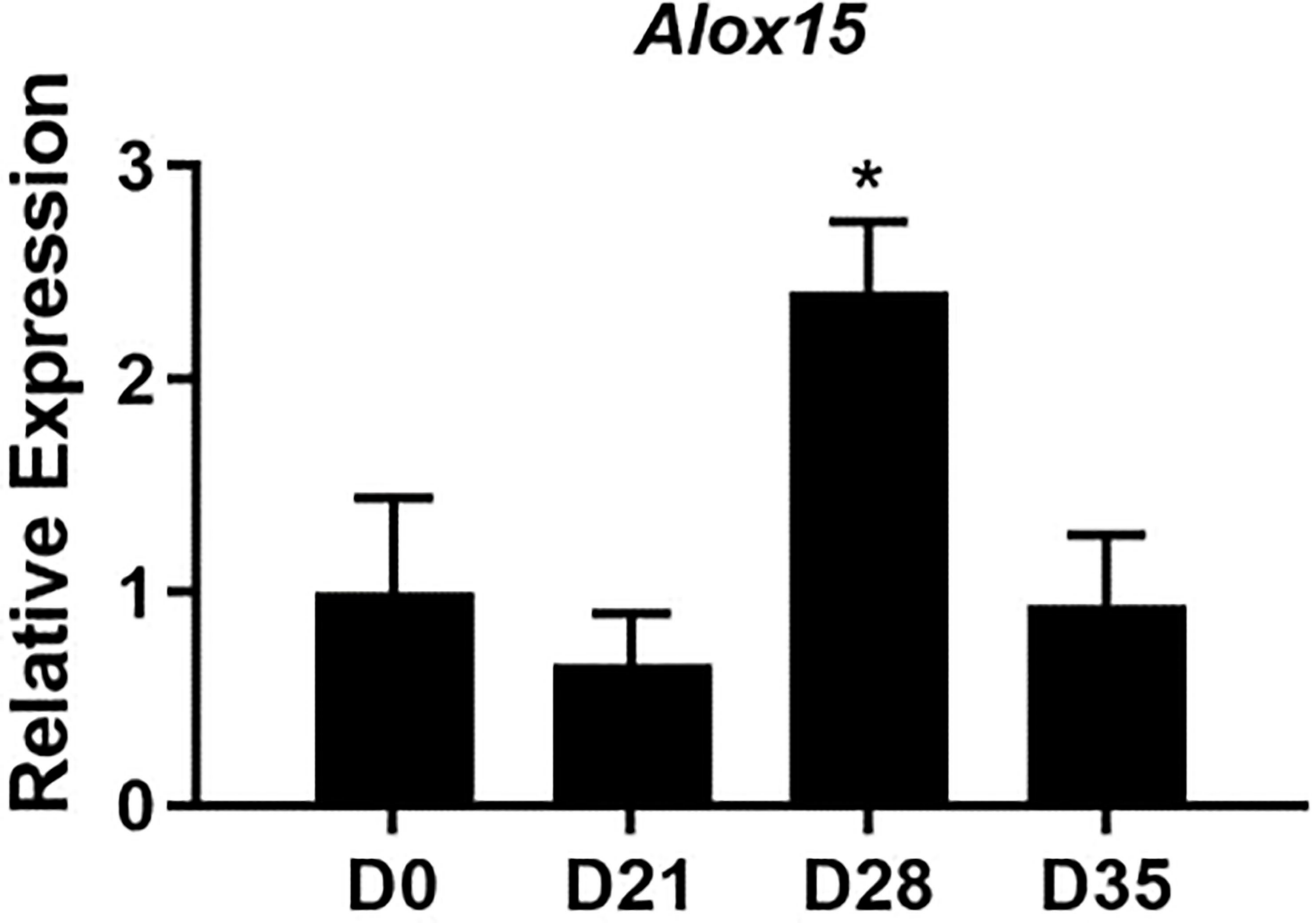
Expression of *Alox15* mRNA over the time-course of mLA. WT C3H mice were infected with *B. burgdorferi* and groups of mice were sacrificed on the days indicated. The expression of *Alox15* mRNA in ankle joints was determined and normalized to *Nid1* and reported relative to D0 (uninfected) levels. n=10/group. *p<0.05 by one way-ANOVA with Dunnett’s test, compared to D0.

**Figure 2:**
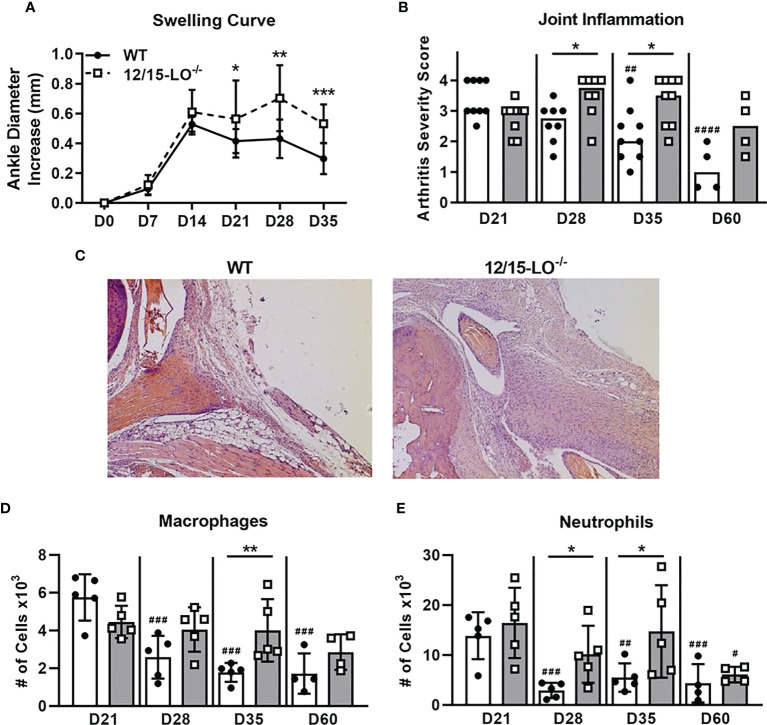
12/15-LO-/- C3H mice display defective mLA resolution. C3H WT and 12/15-LO-/- mice were infected with *B burgdorferi* and mLA was followed over time. **(A)** Ankle swelling curve throughout mLA displayed as diameter change from D0 (uninfected). Closed circles and solid line represent WT mice and open squares and dotted line represent 12/15-LO-/- mice. n=10/group. *p<0.05, **p<0.01, ***p<0.001 by t-test between strains per timepoint. ^##^p<0.01, ^####^p<0.0001 by Kruskal-Wallis one-way ANOVA with Dunnett’s test compared to D21 of the respective strain. **(B)** Arthritis severity scores as determined by histological analysis of H&E-stained ankle joints (scale 0-4) from mice sacrificed on the indicated days. Closed circles represent WT and open squares represent 12/15-LO-/- individual animals. Open bars represent median values from WT mice and grey bars represent 12/15-LO-/- mice. n=4-9/group. Data from two experiments were combined to increase the n value for D21, D28, and D35 groups. *p<0.05 Mann-Whitney U test. **(C)** Representative histology from WT and 12/15-LO-/- ankles at D35. Ankle joints were isolated and **(D)** macrophage (CD45.2+F4/80+) and **(E)** neutrophil (CD45.2+Ly-6Ghi) populations were quantified by flow cytometry. n=4-5/group. *p<0.05, **p<0.01 by t-test between strains per timepoint. ^#^p<0.05, ^##^p<0.01, ^###^p<0.001 by one-way ANOVA with Dunnett’s test compared to D21 of the respective strain.

Lipoxins and specialized proresolving mediators (SPM) are metabolites of 12/15-LO activity that are well-characterized for their ability to mediate resolution in various models of inflammation, including arthritis (27). We first characterized the expression of *Alox15*, the gene which encodes 12/15-LO in mice, during the time-course of mLA. We found that *Alox15* transcript was significantly upregulated in ankle joints of *B. burgdorferi*-infected mice at 28 dpi compared to baseline (0 dpi; **Figure 1**). The timing of this increase suggested a role for 12/15-LO metabolites in the resolution of mLA and we hypothesized that 12/15-LO activity would be required for efficient arthritis resolution. To test this hypothesis, we infected arthritis-susceptible C3H WT and 12/15-LO^-/-^ mice with *B. burgdorferi* and followed arthritis progression. We found that 12/15-LO-deficiency did not affect arthritis development but did impact arthritis resolution. Ankle swelling was similar between the two strains out to day 14 dpi which was near the peak of ankle swelling in WT mice. In the 12/15-LO^-/-^ mice, ankle swelling remained significantly higher throughout the remaining time-course (35 dpi) while joint swelling in the WT mice receded (**Figure 2A**). Comparing arthritis severity scores at specific time points, WT and 12/15-LO^-/-^ scores were similar at day 21 dpi, again suggesting the development of arthritis was similar between the two mouse strains (**Figure 2B**). On days 28 and 35 dpi, however, severity scores in the 12/15-LO^-/-^ joints were significantly higher than WT. On day 60 dpi there were no significant differences in severity scores between the mouse strains although the 12/15-LO^-/-^ scores remained higher even at this late time point. When looking at the severity scores over time it is clear inflammation in the WT mice began to resolve after 21 dpi while in the 12/15-LO^-/-^ joints resolution was significantly delayed. This is readily apparent in the representative histology images from day 35 dpi (**Figure 2C**).

As shown in representative histology (**Figure 2C**), we observed that 12/15-LO^-/-^ mice had elevated cellular infiltrate in arthritic ankles at 35 dpi, even as cellular infiltrate decreased over time in WT mice. We used flow cytometry to quantify innate immune cell infiltrates present in ankles during arthritis progression in 12/15-LO^-/-^ and WT mice (**Figures 2D, E**). Macrophage (CD45.2^+^F4/80^+^) and neutrophil (CD45.2^+^Ly-6G^hi^) populations in ankle tissue peaked around 21 dpi and then significantly declined throughout the remaining time-course in WT mice. However, in 12/15-LO^-/-^ mice, both macrophage and neutrophil populations in the ankle joint were maintained near peak levels, demonstrating a failure of resolution in these animals. Macrophage numbers were significantly higher in 12/15-LO^-/-^ mice compared to WT mice at 35 dpi (**Figure 2D**). Similarly, neutrophil numbers were significantly higher in 12/15-LO^-/-^ mice than WT mice at both 28 and 35 dpi (**Figure 2E**). By 60 dpi, macrophage and neutrophil levels in ankle joints of 12/15-LO^-/-^ mice were again similar to WT, demonstrating a significant delay in the clearance of these innate populations.

### Humoral immune response and control of joint spirochete loads

**Figure 3:**
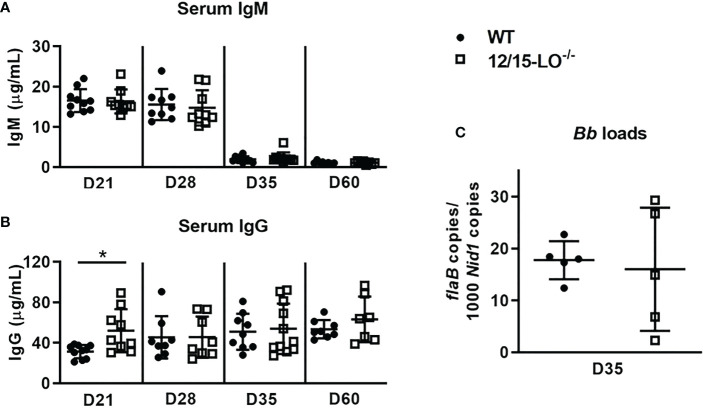
12/15-LO deficiency does not affect the anti-*Borrelia* immune response. C3H WT and 12/15-LO^-/-^ mice were infected with *B. burgdorferi* and sacrificed on the indicated dpi. Blood was collected and *B. burgdorferi*-specific serum IgM **(A)** and IgG **(B)** levels were determined by ELISA. n=7-10/group. *p<0.05 by t-test between strains per timepoint. **(C)** *B. burgdorferi* loads in ankles at D35 by qPCR. n=5/group.

In C3H mice, Lyme arthritis resolution is correlated with spirochete clearance from the ankle joints (9). Therefore, we sought to determine if the defect in efficient arthritis resolution in 12/15-LO^-/-^ mice could be the result of a compromised humoral immune response and poor spirochete clearance from joint tissues. Since antibodies are an important component of spirochete clearance (9), we collected serum from WT and 12/15-LO^-/-^ mice throughout the time-course of mLA and measured *B. burgdorferi*-specific antibody levels. As shown in **Figure 3A**, 12/15-LO^-/-^ mice successfully mounted a humoral response to *B. burgdorferi* infection and produced levels of *Borrelia*-specific IgM no different than WT mice. 12/15-LO^-/-^ mice produced significantly higher levels of *Borrelia*-specific IgG at day 21 dpi, but IgG levels were no different than WT levels at later time points (**Figure 3B**). Lastly, we confirmed that the anti-*Borrelia* humoral response produced by 12/15-LO^-/-^ mice was sufficient to limit *B. burgdorferi* growth in infected ankles to a similar extent as in WT mice (**Figure 3C**). These data suggest 12/15-LO activity has little effect on the development of an anti-*Borrelia* humoral response and that a defect in *B. burgdorferi* clearance from the infected joints is not driving the non-resolution of arthritis in the 12/15-LO^-/-^ mice.

### Defective efferocytosis of apoptotic 12/15-LO^-/-^ neutrophils

**Figure 4:**
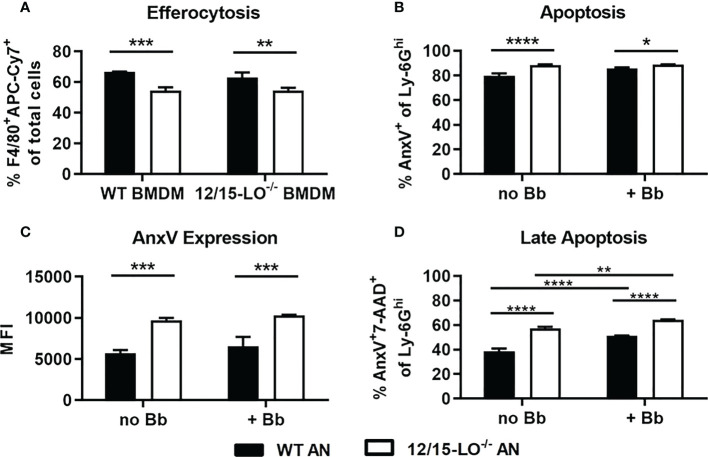
Apoptosis and efferocytosis in 12/15-LO^-/-^ bone marrow neutrophils and macrophages. Bone marrow neutrophils (BMN) were isolated and cultured for 24hr to induce apoptosis. **(A)** Untreated APC-Cy7-stained apoptotic BMN (AN), either WT (black bars) or 12/15-LO^-/-^ (white bars), were cultured with WT or 12/15-LO^-/-^ BMDM (2:1) for 4hr, and efferocytosis, defined as F4/80^+^APC-Cy7^+^ cells, was determined by flow cytometry. Apoptosis of WT and 12/15-LO^-/-^ in 24hr culture +/- *Bb* (MOI 10) was measured by proportion **(B)** and MFI **(C)** of AnxV expression. **(D)** Late-stage apoptosis in cultures from B&C as measured by AnxV and 7-AAD co-staining. n=3/group, assayed in duplicate. **(A–C)** *p<0.05, **p<0.01, ***p<0.001, ****p<0.0001 by t-test, **(D)** one-way ANOVA with Tukey’s posttest.

Efferocytic clearance of apoptotic cells is a critical component of inflammation resolution via both the timely removal of apoptotic cells to prevent secondary necrosis and the reprogramming of efferocytosing macrophages toward a proresolving phenotype (28). As lipoxins and several SPM can promote efferocytosis (29, 30), we hypothesized that clearance of apoptotic neutrophils from joints in 12/15-LO^-/-^ mice could be defective and contribute to the non-resolution of arthritis seen in these mice. Using a reciprocal assay approach, we tested the ability of WT and 12/15-LO^-/-^ BMDM to clear WT or 12/15-LO^-/-^ apoptotic neutrophils (AN). Regardless of BMDM strain, a significantly smaller proportion of BMDMs were able to efferocytose 12/15-LO^-/-^ versus WT AN (**Figure 4A**). WT and 12/15-LO^-/-^ BMDM cleared apoptotic cells similarly, but 12/15-LO^-/-^ AN were cleared less efficiently by both BMDM strains. This result suggests there is an intrinsic difference in 12/15-LO^-/-^ AN that may prevent their efficient clearance from inflammatory sites. We next determined if the lack of 12/15-LO in neutrophils altered their ability to undergo apoptosis and whether this was impacted by the presence of *B. burgdorferi*. Following twenty-four hours in culture, BMN from 12/15-LO^-/-^ mice had a slight but significant increase in their percentage of annexin V-expressing cells regardless of the presence of *B. burgdorferi* (**Figure 4B**). In addition, 12/15-LO^-/-^ BMN also expressed significantly higher levels of annexin V than WT BMN as determined by flow cytometry, again regardless of the presence of *B. burgdorferi* in the cultures (**Figure 4C**). Inefficient clearance of apoptotic cells may increase the number of cells in late-stage apoptosis which may progress into secondary necrosis and prolong inflammation. When WT and 12/15-LO^-/-^ AN were co-stained with 7-AAD as a measure of membrane damage, a higher proportion of 12/15-LO^-/-^ AN than WT AN were AnxV^+^7-AAD^+^, indicating late-stage apoptosis (**Figure 4D**). The presence of *B. burgdorferi* in the culture significantly increased the percentage of cells from both strains in late-stage apoptosis, suggesting the uptake of *B*. *burgdorferi* by neutrophils may induce apoptosis. These findings suggest a failure to efficiently clear apoptotic neutrophils may contribute to the lack of arthritis resolution in 12/15-LO^-/-^ mice.

### *B. burgdorferi* induces LXA_4_ production and mFpr2 transcription from WT BMDM

**Figure 5:**
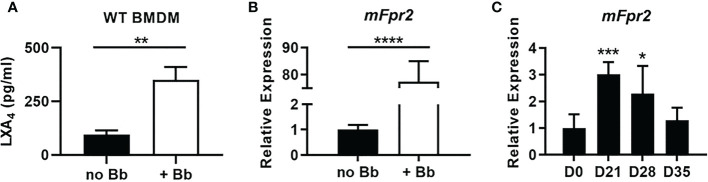
LXA_4_:mFpr2 signaling axis components are upregulated in response to *B. burgdorferi*. **(A)** LXA_4_ production by WT BMDMs after 2hr incubation with control (no *Bb*) or *Bb* (MOI 10). **(B)** *mFpr2* mRNA expression by RT-qPCR after 24hr culture +/- *Bb*, normalized to *Gapdh* and relative to expression in unstimulated (no *Bb*) BMDM. **(C)** *mFpr2* mRNA by RT-qPCR from WT ankle joints during mLA, normalized to *Nid1* and relative to D0 levels. **(A, B)** n=3/treatment, assayed in duplicate, *p<0.05, **p<0.01, ***p<0.001, ****p<0.0001 by t-test. **(C)** n=7-10/timepoint, one-way ANOVA with Dunnett’s posttest, compared to D0.

Metabolic activity by 12/15-LO results in the production of numerous pro-resolution lipid metabolites such as lipoxins, resolvins, and protectins (31). LXA_4_ is an AA-derived metabolite of 12/15-LO enzymatic activity and can promote resolution in other models of arthritis (14, 32–35). LXA_4_ has not previously been isolated out of mLA ankles (36) and may be technically difficult to measure out of tissue due to its rapid metabolism *in vivo* (37). However, we have identified high levels of its metabolic precursors, 12-HETE and 15-HETE, in joints during mLA (36), so we sought to determine if *B. burgdorferi* could induce LXA_4_ production *in vitro*. We cultured WT BMDM with or without *B. burgdorferi* (MOI 10) for 2hr and measured LXA_4_ production (**Figure 5A**). Co-culture of *B. burgdorferi* with BMDM resulted in high levels of LXA_4_ production. Further, transcription of the murine LXA_4_ high-affinity receptor, mFpr2 (38), was significantly upregulated in WT BMDM following co-culture with *B. burgdorferi* (**Figure 5B**). We also confirmed that *mFpr2* transcription is increased during mLA in C3H mice (**Figure 5C**). Together, these findings suggest that *B. burgdorferi* can upregulate components of LXA_4_:mFpr2 signaling, providing a rationale to investigate the ability of LXA_4_:mFpr2 signaling to resolve inflammation in mLA.

### Exogenous LXA_4_ reduces ankle edema during mLA in WT mice

**Figure 6:**
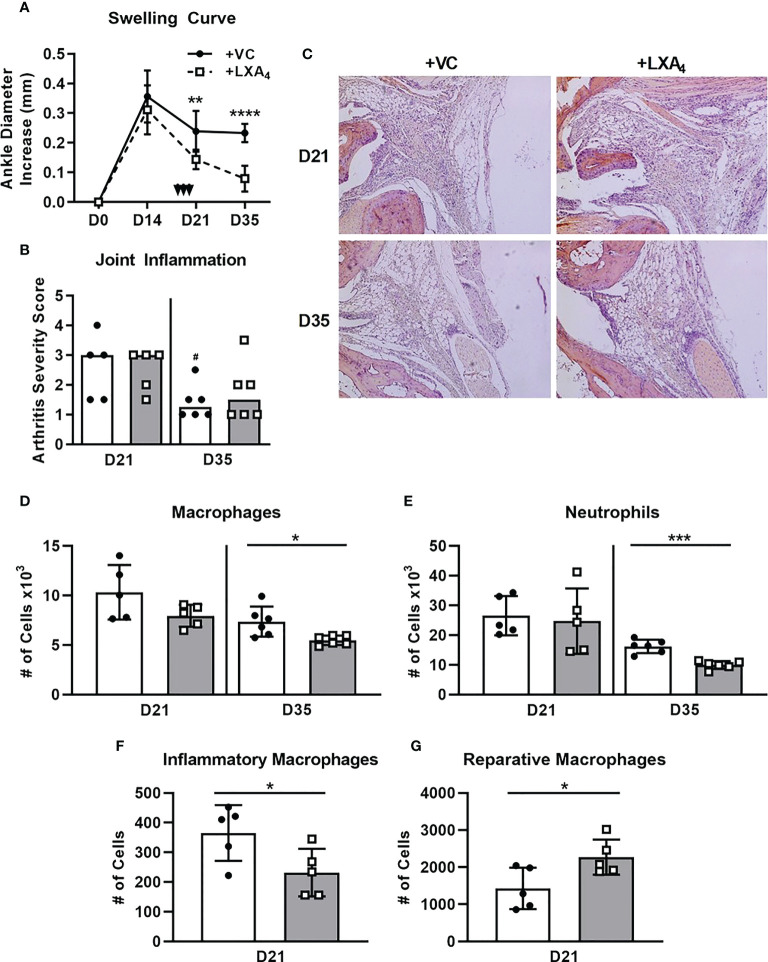
Exogenous LXA4 treatment reduces ankle edema and accelerates joint inflammatory cell removal. C3H WT mice were infected with *B. burgdorferi* and treated with either vehicle control (VC; closed circles, solid line) or LXA4 (open squares, dotted line) i.p. on days 18,19, and 20pi (arrows). Development of mLA was followed over time. **(A)** Ankle diameters were measured on the days indicated. n=5/group. **p<0.01, ****p<0.0001 by t-test between treatment groups per timepoint. **(B)** Arthritis severity scores of H&E-stained ankle joints from mice sacrificed on D21 and D35. VC (closed circles, open bars), LXA4 (open squares, grey bars). Bars represent median values. n=5-6/group. ^#^p<0.05 by Mann-Whitney U test compared to D21 of the respective strain. Representative histology is in **(C)**. Total macrophages **(D)** and neutrophils **(E)** quantified from the ankle joints of VC- (closed circles, open bars) or LXA4-treated (open squares, closed bars) WT mice at D21 and D35 by flow cytometry. Two macrophage subsets were further measured in D21 ankles: inflammatory macrophages **(F;** CD45.2+F4/80+Ly-6Chi) and reparative macrophages **(G;** CD45.2+F4/80+Ly-6Cint/lo). n=5/group. *p<0.05 by t-test between treatment groups per timepoint. Data from two experiments were combined to reach power in D35 groups **(A, B, D, E)**. ***p<0.001.

Previous studies in other murine arthritis models have had varied results when investigating the therapeutic efficacy of exogenous LXA_4_. For example, one study on zymosan-induced arthritis in mice found that LXA_4_ administration ameliorated inflammation by reducing edema and leukocyte infiltration (35). However, another study using a *S. aureus*-induced septic arthritis mouse model found that treatment with LXA_4_ impaired inflammation resolution because it interfered with pathogen control (39). Therefore, while we hypothesize that exogenous LXA_4_ will ameliorate mLA in WT C3H mice, it is important to avoid interfering with bacterial clearance to prevent prolonged active infection and inflammation amplification. To investigate the therapeutic capacity of exogenous LXA_4_ for mLA in WT mice, we infected WT C3H mice with *B. burgdorferi* to induce mLA, then administered either a vehicle control (VC) or 1μg LXA_4_ i.p. per day on 18, 19, and 20 dpi and measured resolution outcomes. As shown in **Figure 6A**, treatment with LXA_4_ resulted in an acute reduction of edema compared to VC-treated mice, starting at 21 dpi and continuing through 35 dpi.

To determine if the reduction in edema in LXA_4_-treated mice correlated to a decrease in inflammation at these timepoints, we performed histological scoring of ankle sections at both 21 and 35 dpi and found that VC- and LXA_4_-treated mice had similar arthritis severity at both timepoints (**Figures 6B, C**). We also confirmed that LXA_4_ delivery at this timepoint did not interfere with formation of an anti-*Borrelia* immune response, with both *B. burgdorferi*-specific IgM and IgG successfully produced and *B. burgdorferi* as efficiently cleared in VC- and LXA_4_-treated mice (**Supplementary Figure 1**). These data demonstrate that while joint edema was significantly reduced by LXA_4_ treatment, this strategy of LXA_4_ delivery did not ameliorate underlying inflammatory responses by 35 dpi. In addition, we found no differences between the absolute number of macrophages or neutrophils in VC- and LXA_4_-treated mice at 21 dpi (**Figures 6D, E**); however, there were significantly fewer macrophages and neutrophils in LXA_4_-treated joints at 35 dpi. Although overall macrophage numbers were not different between treatment groups at 21 dpi, we hypothesized that LXA_4_ may have altered the inflammatory phenotypes of the macrophage population, since it is known to induce non-phlogistic monocyte recruitment to assist in inflammation resolution (40). To test this hypothesis, we measured Ly-6C expression on F4/80^+^ cells as an indication of inflammatory state, with F4/80^+^Ly-6C^hi^ cells categorized as inflammatory macrophages and F4/80^+^Ly-6C^int/lo^ cells categorized as reparative macrophages (41). In doing so, we found that LXA_4_-treated mice had significantly fewer Ly-6C^hi^ inflammatory macrophages and significantly more Ly-6C^int/lo^ reparative macrophages compared to VC-treated mice (**Figures 6F, G**). Together these data demonstrate that LXA_4_ treatment significantly reduced joint edema, promoted a reparative macrophage phenotype, and accelerated the removal of inflammatory cells from the joints by 35 dpi.

## Discussion

Following acute inflammation, timely resolution is required to recover homeostasis and prevent further tissue damage. To promote the switch to a pro-resolution program, several endogenous mediators are released at the site of inflammation, including metabolites downstream of 12/15-LO such as lipoxins and SPM. These pro-resolving lipids act through a variety of mechanisms, including inhibition of inflammatory leukocyte recruitment, induction of neutrophil apoptosis and efferocytosis, and reprogramming of macrophages towards a pro-resolving phenotype (42). Previous studies demonstrate a clear role for lipoxins and SPM in regulating inflammation in degenerative or autoimmune arthritis in humans and in mouse models of arthritis (27). Studies in rheumatoid arthritis (RA) patients demonstrated that lower levels of circulating SPM could clearly distinguish active RA patients from healthy controls, indicating a correlation between SPM expression and disease status (32, 43). Further, elevated synovial 15-LO expression in active RA patients (44) was positively correlated with upstream prostaglandin E_2_ signaling and downstream LXA_4_ production in synovial fluid (45), implicating LXA_4_ as a negative feedback mechanism for proinflammatory mediator production in RA. In mice, a 12/15-LO deficiency resulted in exacerbated arthritis in the K/BxN serum transfer arthritis model, with increased proinflammatory gene expression and reduced LXA_4_ levels in affected joints (33). Using this same arthritis model, prophylactic treatment with 17*R*-resolvin D1 (17*R*-RvD1) or resolvin D3 (RvD3) improved arthritis clinical scores, reduced edema, and inhibited inflammatory leukocyte migration (32, 34). Similarly, treatment with exogenous LXA_4_ promoted resolution in a zymosan-induced arthritis model by reducing edema formation and joint leukocyte infiltration (35). These studies reveal a critical role for 12/15-LO products in resolution of joint inflammation and demonstrate that treatment with lipoxins or SPM may improve arthritis outcomes.

Here, we sought to determine the role of 12/15-LO and downstream metabolites in the development and resolution of mLA. Despite the known presence of several 12/15-LO metabolites during arthritis development (36), 12/15-LO^-/-^ C3H mice were capable of developing mLA to a similar extent as WT C3H mice, indicating that proinflammatory metabolites downstream of 12/15-LO are not required for mLA development. On the other hand, 12/15-LO^-/-^ mice did not readily resolve mLA, retaining significant ankle edema and inflammatory infiltrate even as WT mLA resolved. These results confirm that proresolving lipids downstream of 12/15-LO activity are required for mLA resolution.

In WT C3H mice infected with *B. burgdorferi*, spontaneous resolution of mLA coincides with the clearance of bacteria from the ankle (9). However, we have previously demonstrated that neither COX-2^-/-^ nor 5-LO^-/-^ mice resolve mLA despite equivalent bacterial control to WT mice, indicating a critical role for metabolites downstream of both COX-2 and 5-LO in mLA resolution (11, 12). Here we report a similar finding in 12/15-LO^-/-^ mice, where bacterial burdens are controlled to the same extent as in WT mice, but arthritis is not resolved. As COX-2 products, 5-LO, and 12/15-LO can cooperate in lipoxin and SPM synthesis (8, 15, 16), the nonresolution phenotype seen in COX-2^-/-^, 5-LO^-/-^, and 12/15-LO^-/-^ mice despite spirochete clearance from the joint may be due to a shared defect of lipoxin and SPM production. We are currently investigating these possibilities further.

Lipoxins and SPM can promote macrophage clearance of apoptotic cells in the inflamed site, during which macrophages can be remodeled towards a proresolution phenotype through a positive feedback loop (29, 30, 42). This key step in inflammation resolution also ensures that apoptotic cells do not progress towards necrosis, during which they release their cellular components as DAMPs and thereby amplify inflammation (28). Here we report that 12/15-LO^-/-^ ankle joints had persistent macrophage and neutrophil infiltration at 28 and 35 dpi, despite clearance of these populations in WT mice. Subsequent *in vitro* experiments suggested that the prolonged presence of inflammatory cells in 12/15-LO^-/-^ mice could be due to a defect in the clearance of 12/15-LO^-/-^ AN. However, a larger proportion of 12/15-LO^-/-^ AN in culture underwent apoptosis, even expressing more of the key “eat me” signal phosphatidylserine by proportion of culture and MFI than did WT AN under similar conditions. These findings indicate that there was not a defect in the level of apoptosis or the expression of phosphatidlyserine in 12/15-LO^-/-^ AN cultures, so it may be a secreted “find me” factor, or lack thereof, from 12/15-LO^-/-^ AN that hampered their efferocytosis by macrophages. In addition, as LXA_4_ and several resolvins are also known for their ability to inhibit neutrophil extravasation into the site of inflammation (46, 47), it may be possible that 12/15-LO^-/-^ mice lacking these mediators could have unchecked neutrophil extravasation into the site of inflammation, although more work needs to be done on this subject. Overall, these findings implicate 12/15-LO and downstream products in efficient efferocytosis of apoptotic neutrophils, and a breakdown in this mechanism in 12/15-LO^-/-^ mice may explain persistent neutrophil and macrophage populations and exacerbated arthritis at WT resolution timepoints.

Studies in a variety of inflammatory disease models have investigated the therapeutic efficacy of lipoxins and SPM, alone and in combination with other treatments (48, 49). Here we sought to determine if exogenous LXA_4_ alone could hasten mLA resolution in WT mice if delivered near the peak of inflammation. In agreement with previous studies (50, 51), we saw that LXA_4_ reduced ankle edema within days of its administration, and this decrease was maintained for at least the next two weeks. LXA_4_ treatment also acutely increased the number of reparative macrophages and reduced the number of inflammatory macrophages in the joint compared to control mice. This is in line with known effects of LXA_4_ signaling through mFpr2 in mice to reprogram macrophages towards a pro-resolution phenotype (52). Further, as exogenous LXA_4_ can either impair or enhance bacterial control depending on the sepsis model (39, 53), we confirmed that exogenous LXA_4_ did not interfere with the formation of an anti-*Borrelia* antibody response and control of spirochete numbers in the joint.

Despite the observed effects of LXA_4_ on edema and cellular infiltrate into the WT mLA joint, the chosen treatment regimen was not sufficient to hasten arthritis resolution by 35 dpi, at which point mLA was nearly resolved in both the VC- and LXA_4_-treated mice. Improvements to the treatment regimen could include direct intra-articular injection of LXA_4_ and repeated, frequent doses of LXA_4_ until resolution. Further, as its rapid degradation causes LXA_4_ to lose biological activity (37), several stable LXA_4_ analogs and synthetic LXA_4_ mimetics have been designed which maintain the proresolving actions of native LXA_4_ (47, 48). Indeed, LXA_4_ mimetic AT-01-KG has been shown to ameliorate joint inflammation in mouse models of gout and adjuvant-induced arthritis (54), so using these LXA_4_ analogs may yield better results in mLA than LXA_4_. Lastly, as endogenous lipid mediators work in concert to resolve inflammation, a combinatorial approach whereby LXA_4_ is delivered with another SPM may further improve mLA outcomes.

In conclusion, we have demonstrated that 12/15-LO is protective in mLA, as 12/15-LO^-/-^ mice do not resolve arthritis in a timely manner despite sufficient bacterial control. This unresolved inflammation may be due in part to a defect in efferocytosis of apoptotic neutrophils in the arthritic joint. As *B. burgdorferi* induced LXA_4_ production and mFpr2 transcription in BMDM and mFpr2 transcription *in vivo*, we investigated the therapeutic ability of LXA_4_ to potentiate mLA resolution. LXA_4_ treatment significantly reduced ankle edema, remodeled macrophage populations in the joint towards a proresolution phenotype, and accelerated the removal of inflammatory cells from the joints. These findings illustrate the importance of bioactive lipid mediator class-switching in a murine model of Lyme arthritis and support the investigation of lipoxins and SPM as potent therapeutic options for Lyme arthritis in humans that can reduce pain and arthritis edema without compromising bacterial clearance from tissues.

## Data availability statement

The raw data supporting the conclusions of this article will be made available by the authors, without undue reservation.

## Ethics statement

The animal study was reviewed and approved by The Animal Care and Use Committee of the University of Missouri.

## Author contributions

CJ and CB conceived and designed the study. CJ performed most of the experiments and KH provided data for one figure. CJ collected the data. CJ and CB scored the histology sections and interpreted all data. CJ wrote the draft of the manuscript and CB completed the final editing. All authors critically discussed the data interpretations and reviewed the final manuscript. All authors contributed to the article and approved the submitted version.
