# Supplementary material for: 12/15-lipoxygenase activity promotes efficient inflammation resolution in a murine model of Lyme arthritis
Source: Front Immunol. 2023 Apr 18;14:1144172. doi: 10.3389/fimmu.2023.1144172 (PMC10151577; doi:10.3389/fimmu.2023.1144172)
Supplement: Supplementary file 1 [file DataSheet_1.pdf]

Supplemental Data

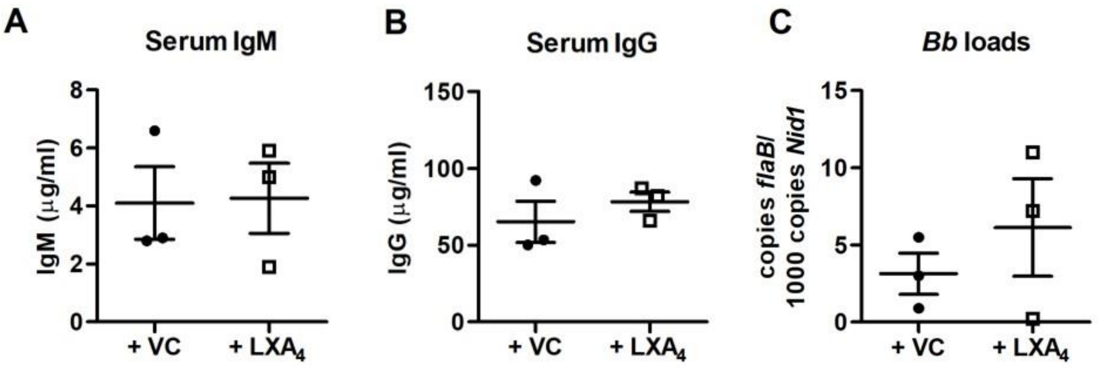

Supplementary Figure 1. Exogenous LXA_4 treatment during mLA does not interfere with the host anti-*Borrelia* response. WT C3H mice were infected with *B. burgdorferi* and were treated with either vehicle (VC) or LXA_4 i.p. on days 18, 19, and 20pi. Mice were sacrificed on D35 and *B. burgdorferi*-specific serum IgM (A) and IgG (B) was determined. (C) *B. burgdorferi* loads from VC or LXA_4 ankles at D35 by qPCR. n=3/group.

Supplementary Table 1. Primers used for qPCR.

Gene	Primer sequence	
B. burgdorferi flaB	F	5'- TCT TTT CTC TGG TGA GGG AGC T- 3'
	R	5'- TCC TTC CTG TTG AAC ACC CTC T- 3'
Mouse *Nid1*	F	5'- AGG GCA GAA TGC CTG AAC C- 3'
	R	5'- AGG ATA CTG GAG CCC TTC GAG- 3'
Mouse *Alox15*	F	5'- GCG ACG CTG CCC AAT CCT AAT C- 3'
	R	5'- CAT ATG GCC ACG CTG TTT TCT ACC- 3'
Mouse *Gapdh*	F	5'- GTG GAC CTC ATG GCC TAC AT- 3'
	R	5'- GGG TGC AGC GAA CTT TAT TG- 3'
Mouse *mFpr2*	F	5'- CTG AAT GGA TCA GAA GTG GTG G- 3'
	R	5'- CCC AAA TCA CTA GTC CAT TGC C- 3'
